# 3D/2D model-to-image registration by imitation learning for cardiac procedures

**DOI:** 10.1007/s11548-018-1774-y

**Published:** 2018-05-12

**Authors:** Daniel Toth, Shun Miao, Tanja Kurzendorfer, Christopher A. Rinaldi, Rui Liao, Tommaso Mansi, Kawal Rhode, Peter Mountney

**Affiliations:** 1 Siemens Healthineers, Frimley, UK; 2 Siemens Healthineers, Medical Imaging Technologies, Princeton, NJ USA; 3 Department of Cardiology, Guys and St. Thomas Hospitals NHS Foundation Trust, London, UK; 4 School of Biomedical Engineering and Imaging Sciences, King’s College London, London, UK; 5 Siemens Healthineers, Forchheim, Germany

**Keywords:** Image fusion, Cardiac registration, Imitation learning, Deep learning, Cardiac resynchronization therapy

## Abstract

**Purpose:**

In cardiac interventions, such as cardiac resynchronization therapy (CRT), image guidance can be enhanced by involving preoperative models. Multimodality 3D/2D registration for image guidance, however, remains a significant research challenge for fundamentally different image data, i.e., MR to X-ray. Registration methods must account for differences in intensity, contrast levels, resolution, dimensionality, field of view. Furthermore, same anatomical structures may not be visible in both modalities. Current approaches have focused on developing modality-specific solutions for individual clinical use cases, by introducing constraints, or identifying cross-modality information manually. Machine learning approaches have the potential to create more general registration platforms. However, training image to image methods would require large multimodal datasets and ground truth for each target application.

**Methods:**

This paper proposes a model-to-image registration approach instead, because it is common in image-guided interventions to create anatomical models for diagnosis, planning or guidance prior to procedures. An imitation learning-based method, trained on 702 datasets, is used to register preoperative models to intraoperative X-ray images.

**Results:**

Accuracy is demonstrated on cardiac models and artificial X-rays generated from CTs. The registration error was $2.92\pm 2.22\,\hbox { mm}$ on 1000 test cases, superior to that of manual ($6.48\pm 5.6\,\hbox { mm}$) and gradient-based ($6.79\pm 4.75\,\hbox { mm}$) registration. High robustness is shown in 19 clinical CRT cases.

**Conclusion:**

Besides the proposed methods feasibility in a clinical environment, evaluation has shown good accuracy and high robustness indicating that it could be applied in image-guided interventions.

**Electronic supplementary material:**

The online version of this article (10.1007/s11548-018-1774-y) contains supplementary material, which is available to authorized users.

## Introduction

Minimally invasive cardiac interventions, such as cardiac resynchronization therapy (CRT), are performed under X-ray fluoroscopy guidance. X-ray imaging is ideal to visualize dense structures; soft tissue contrast is, however, highly limited. In such interventions, preoperative data can be fused with intraoperative images to support interventional navigation. To provide clinically useful fused images, a reliable registration is required. Registering two datasets acquired with fundamentally different imaging modalities (i.e., MR and X-ray) is highly challenging: Intensities, contrast levels and fields of view (FOVs) can be significantly different, and the same structures may not be visible in both modalities.

In CRT, as in most cardiac procedures, these differences can be drastic: The preoperative data are a non-contrast-enhanced MR acquisition, and the intraoperative data are X-ray fluoroscopy. The preoperative MR acquisition is often a short axis stack of cine images, showing soft tissue with high in-plane (1–2 mm), but low out-of-plane resolution (10 mm). The images are highly cropped, the FOV is concentrated on the ventricles, showing only a few surrounding structures. Structures that may be useful for registration such as the spine or the ribs are not visible. On the contrary, fluoroscopy shows dense structure, such as bones or instruments, has high resolution, and can have a much larger FOV.

In the challenging research area of 3D/2D registration, a number of approaches have been proposed [10]. Clinical experts usually register preoperative models or 3D images to 2D fluoroscopy manually [13]. Experts can combine anatomical knowledge with extensive experience of interpreting X-ray images and projective geometry, this is, however, time-consuming, has a learning curve, and is highly user-dependent. Manual registration can be simplified by using fiducial markers [4, 13]. Fiducials can also be used in optical tracking-based registration [14, 15], but these approaches require the preoperative scan to be acquired directly before the intervention and changes to the clinical workflow need to be introduced, which are often not feasible. Manual registration can be supported by tools inserted into veins or cardiac chambers [2]; however, these can distort the anatomy, thus reducing registration accuracy and robustness. Automatic approaches were also developed exploiting endovascular tools [3]. These approaches require a high-resolution preoperative MR scan, to extract endovascular models, that is often not available. Approaches extracting models from SPECT images exist for CRT planning [19]. The models of the left ventricle (LV) are registered to the coronary veins, reconstructed from contrasted X-ray fluoroscopy, by identifying grooves on the surface of the LV. Due to the nature of SPECT imaging (low resolution), this can only be done with multiple assumptions and limited accuracy.

A notable approach specifically designed for CRT relies on adjacent anatomical structures [16, 17]. Similarly to the SPECT-based approaches, the coronary venous anatomy from fluoroscopy is registered to preoperative models of the LV from MR imaging. The method is, however, limitedly applicable, if the contrasted X-ray acquisitions do not have sufficient quality, due to the anatomy, or the contrast injection. The model-based property enables the usage of this method with any preoperative modality, if the required LV model can be extracted.

Learning-based approaches that can be used for guiding procedures were also developed in recent years. A notable approach registers a CAD model of an implant to X-ray images by a convolutional neural network (CNN) regression model [12]. The approach, however, is difficult to generalize to anatomical data; it is only applicable to highly stiff objects of certain shapes, i.e., the implants. The rendering of the implant model is performed similarly to a previous approach [7].

Classical approaches often have low robustness and capture range. Uniform data and a good initial alignment are required. More novel machine learning-based approaches can overcome these challenges. An artificial intelligence-based (AI based) approach was shown to perform rigid 2D/2D and 3D/3D registration robustly on medical data [9]. In this approach, an artificial agent, modeled by an artificial neural network (ANN), is trained to learn a policy, an optimal strategy to take actions depending on the input images. Due to the high robustness of the approach, it is ideal to be applied in interventional guidance, where robustness may be more valuable than accuracy. The approach was extended to solve 3D/2D registration of the spine in CT and fluoroscopy [11]. However, in this approach, the agent takes DRR as input. DRRs can only be rendered for CT. The approach is not directly applicable to registration problems where the 3D modality is MR.

There are two significant challenges in AI-based cross-modality registration: (1) They require large sets of training data with ground truth (GT) registration and (2) they only work on the specific modalities and acquisition protocols they were trained on. The former is a significant problem for CRT. Interventional fluoroscopy is not, in general, automatically stored, patients may be imaged in modality-specific positions (e.g., arms up / arms down) causing a non-rigid transformation, and manually generating GT registration is time-consuming and inaccurate. The latter makes the registration systems vulnerable to changes in acquisition protocols and prevents general adoption of the same system for multiple clinical procedures.

In the pursuit of a general and robust cross-modality registration framework, this paper exploits a byproduct of the preoperative diagnostic process—anatomical models. In order to diagnose or characterize diseases, it is common to segment the anatomy of interest (i.e., LV for CRT). The main advantage of using preoperative models is that the registration framework can be generalized, as it is independent of preoperative voxel intensities and acquisition parameters. The method can be trained on a single modality and applied to other modalities representing the same anatomy without retraining for specific cross-modality images.

In this paper, a novel solution for multimodality registration for cardiac procedures is presented that has minimal interference with standard clinical routine. The approach is a combination of 3D model extraction from preoperative data and an artificial intelligence-based registration framework [11]. The system is capable of registering preoperative models, without relying on voxel intensities or features from the preoperative modality, to a single 2D X-ray image. This means that the preoperative data can be of any modality (e.g., MR, CT or ultrasound), if relevant models can be extracted. The approach requires a single X-ray acquisition; thus, the clinical workflow does not need to be amended to acquire a second image. As a further advantage, since preoperative models are often created during preoperative planning and diagnostic reporting, the model extraction is not an additional complication. Thus, the method can provide a robust registration for interventional guidance, without major interference with the standard clinical workflow.

## Materials and methods

### Overview

**Fig. 1:**
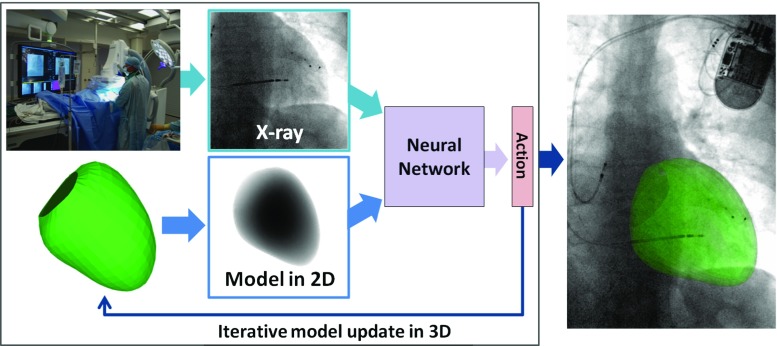
Overview of the model-to-image registration method with an artificial agent

The idea is to register models extracted from preoperative data, i.e., MR to intraoperative X-ray fluoroscopy, to guide cardiac interventions. An overview with a trained agent is illustrated in Fig. 1. The 3D preoperative data are segmented prior to the intervention, to extract a model of the anatomy of interest, i.e., the LV. During the intervention, an X-ray image (the fixed image) is acquired. A 2D projection image of the LV model is generated (the moving image) with the same imaging geometry as the X-ray image. The two images are shown to an agent, modeled by an ANN, that predicts the reward (the better the direction of an action, the higher the reward) for each possible action. The action with the maximum reward is chosen and is applied to the 3D model. The moving image is regenerated from the transformed model. These steps are iteratively repeated until convergence.

In the current setup, the registration is performed between a 3D model and a single fluoroscopy frame, not accounting for cardiac and respiratory motion in consecutive frames. The depth is assumed to be approximately correct after isocentering the volume and the X-ray image. The registration problem is restricted to the 3 degrees of freedom (DOF) in the imaging plane: *x* (horizontal) and *y* (vertical) translation and a rotation (around the axis of projection *z*).

### Imitation learning

The registration task can be formulated as a type of reinforcement learning problem [9], imitation learning. The agent’s steps can be modeled as a Markov decision process: $\{ S, A, \tau , r, \gamma \}$, where *S* represents the possible states, *A* the possible actions, $\tau$ is the probability of an action taken from a state at a certain time step, *r* is the reward for an action, and $\gamma$ is the discount factor, defining the importance of long-term rewards. The agent is in a single state (alignment) $s_t$ at a certain time step *t* and actions (steps) $a_t$ in every direction along each DOF are rewarded, depending on the effectiveness (better or worse alignment) of action $a_t$. The goal is to learn a policy $\pi$, an optimal registration strategy, that can predict the optimal action with the highest reward from the current state $S_t$:

1 $$\begin{aligned} a_t = \pi (S_t), \end{aligned}$$

thus to maximize the long-term reward:

2 $$\begin{aligned} \sum _{t=0}^{\infty } \gamma ^t r_{a_t}, \end{aligned}$$

where $r_{a_t}$ is the reward for action $a_t$. The agent can be modeled by an artificial neural network (ANN) and by training, a policy is learned by the network. The policy will imitate what the agent was being shown during training.

The agent is trained in a supervised manner: It is shown two images in the current state and the optimal rewards. The rewards are defined in a way that an action receives a higher reward, if it brings the moving image closer to the optimal alignment. The improvement, thus the reward $r_{t+1}$, is defined as the difference of distances between the old transformation $T_t$ and GT transformation $T_g$, and the current transformation $T_{t+1}$ and the GT transformation:

3 $$\begin{aligned} r_{t+1} = D(T_g, T_t) - D(T_g, T_{t+1}). \end{aligned}$$

The distance between two transformations $T_1$ and $T_2$ is $D(T_1, T_2)$, the L2 norm of the parameters of the transformations, as described in [9].

### Architecture

**Fig. 2:**
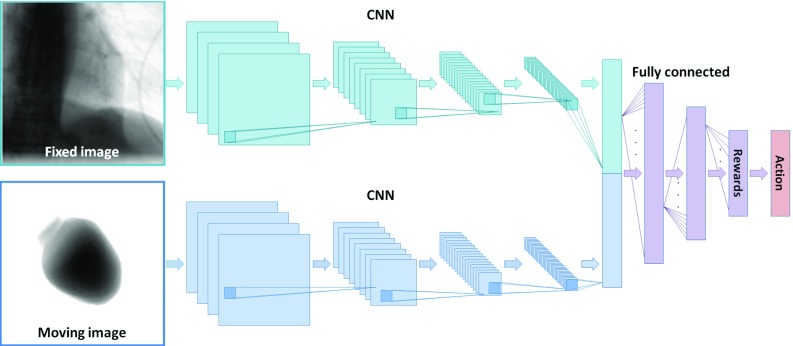
Architecture of the neural network that represents the artificial agent

The agent is modeled by a pair of CNNs to encode the input images into features and another neural network (NN) that decodes the features to determine the rewards, see Fig. 2. The input layer of each CNN is defined to be $128\times 128$, and the input images are resampled to match this resolution. The CNNs consist of 4 convolutional layers, each followed by rectified linear units (ReLU) and a max-pooling layer. Batch normalization was applied after each layer. The CNNs result in feature vectors that represent the data. The feature vectors are concatenated and a NN with 4 fully connected layers, followed by ReLU layers and batch normalization, decodes the feature vectors to predict the rewards.

### Model-to-image registration

To train an agent for registration, perfectly aligned 3D models and 2D images are required. It is highly challenging to have a GT registration for MR or US to X-ray data and, additionally, the number of available multimodal datasets is highly limited. Due to these reasons, only CT images are used for training: The 3D models are extracted by segmentation, and the 2D images are generated by projections.

**Fig. 3:**
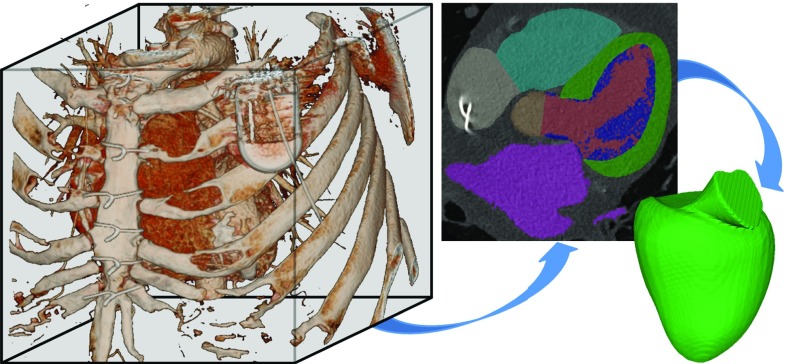
Model extraction from CT images

The CT datasets were automatically segmented by a combination of object localization and a multistep non-rigid deformation estimation [18]. The segmentation results in a binary mask and a mesh model of the LV, see Fig. 3.

#### 3D/2D registration model

The problem of different dimensionalities was solved as in [11]; 2D images were shown to the agent. The fixed and moving images for every training sample are generated from the same CT dataset. The fixed image is a digitally reconstructed radiograph (DRR) [8] representing the intraoperative X-ray image. The DRRs are projection images of the CT volume, based on the X-ray attenuation model. The center of projection is defined to be the center point of the LV model. The fixed image was generated with a large FOV, i.e., $300\,\hbox { mm}\times 300\,\hbox { mm}$. The moving image, the projection of the LV model, was generated with a smaller FOV, i.e., $120\,\hbox { mm}\times 120\,\hbox { mm}$, having the LV centered. The model projection image will correspond to a subregion of the fixed image, the region of interest (ROI). Translation is performed by moving the LV model in 3D and regenerating the moving image, while keeping the LV model in the center of the FOV. This way, for consecutive translations, the projection image will correspond to different subregions of the fixed image. The ANN, modeling the agent, is shown an image pair, the moving image and the corresponding ROI extracted from the fixed image.

#### Training setup

The LIDC-IDRI public dataset [1] and previously acquired data were used (802 contrasted volumes). The data were split to 702 training and 100 test datasets. To generate a sufficient number of training pairs, the 702 training datasets were augmented. This was mainly performed by perturbing the perfectly aligned, generated image pairs. Transformations with the 3 DOF (2 translations and 1 rotation), defined by the imaging plane of the fixed image, were applied to the 3D mask.

The maximal perturbation of the translation components of the GT transformation was 35 mm, and the maximal rotation component was ${15}^{\circ }$. These values correspond to misalignment observed after the isocenters of an MR volume and a fluoroscopy image are co-registered. Furthermore, a random, maximally 10 mm offset to the center of projection was introduced, since the heart is not perfectly centered in fluoroscopy acquisitions. Additionally, the primary positioner angulation (left/right anterior oblique) was varied between $-\,{15}^{\circ }$ and $+\,{15}^{\circ }$, and the secondary angle (caudal/cranial) between $-\,5^{\circ }$ and $+\,5^{\circ }$. By generating 1000 perturbations for each of the 702 training datasets, 702,000 perturbations were created.

The network described in the “Architecture” section was trained with a minibatch size of 80. The solver used was RMSProp with a momentum of 0.9, and the learning rate was 0.01 with a decay ratio of 0.8 after every 10,000 iterations. Training took about 20 h on an NVIDIA GeForce GTX Titan X Pascal GPU.

## Evaluation and results

### Synthetic data

As described in the “Training setup” section, the data were split into 702 training and 100 test datasets. The registration performance was evaluated qualitatively and quantitatively, by perturbing each test dataset 10 times, resulting in 1000 test cases.

#### Qualitative evaluation

**Fig. 4:**
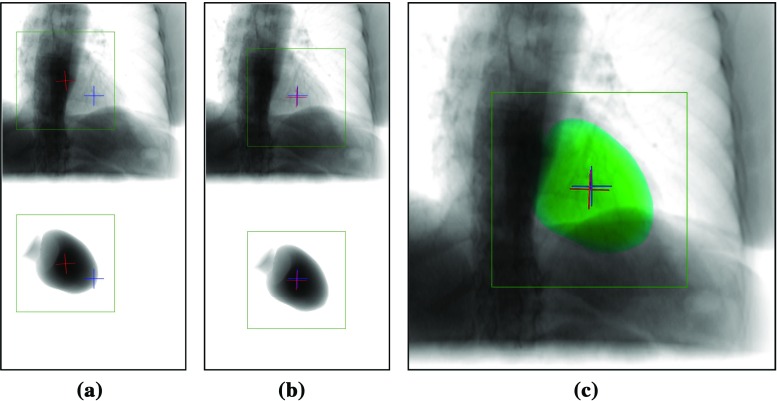
Relation of fixed and moving image **a** before, **b** after registration, and **c** the overlay of the registered mask (green). Showing the ROI (green box), the fixed (blue cross) and the moving image landmark (red cross)

To evaluate the method qualitatively, the projections of the LV model were compared with the corresponding fluoroscopy images after registration, see Fig. 4. The only visual cue inherently found in the fluoroscopy image is the shadow of the left ventricle. Additionally, a cross-shaped landmark is defined at the center of the LV, computed from the model of the LV. The cross extends 10 mm from the center point. In successful registrations, the shadow of the left ventricle in fluoroscopy matches the border of the projected LV model and the landmarks are located at the same location, having the same orientation, in both images, see Fig. 4c.

#### Quantitative evaluation

The target registration error (TRE) was measured by computing the L2 norm of the points of the cross landmark at the center, described in “Qualitative evaluation” section, between the GT fluoro cross (blue) and the registered LV model cross (red), see Fig. 4. The TRE was computed in 2D, because the registration is performed in-plane, the depth is not adjusted, thus the 3D error would not provide more information.

The method was evaluated against manual and gradient-based automatic registration, see Table 1. The gradient-based metrics were gradient correlation (GC), gradient information (GI) and gradient orientation (GO) [5] and their versions utilizing only the positive gradients (GC+, GI+, GO+), corresponding to the visible heart shadow in the images. The agent’s results were significantly better than those of the other approaches. The starting TRE of $22.8\pm 10.5\,\hbox { mm}$ was improved to $2.92\pm 2.22\,\hbox { mm}$, the median TRE was reduced from 21.42 to 2.34 mm, and the angular error from $7.17^{\circ }\pm 4.64^{\circ }$ to $2.34^{\circ }\pm 1.87^{\circ }$. The best gradient-based method, GI+, has resulted in a TRE of $6.79\pm 4.75\,\hbox { mm}$ with a median of 5.63 mm and an angular error of $7.28^{\circ }\pm 4.71^{\circ }$. Showing slightly lower accuracy than manual registration (mean: $6.48\pm 5.60\,\hbox { mm}$, median: 4.93 mm, angle: $6.21^{\circ }\pm 5.17^{\circ }$).

The main reason for failures in gradient-based methods was that the highest metrics score is at the liver dome or the spine, providing the strongest gradients in the DRRs. The amended methods (GO+, GC+, GI+) counteract this, by using only positive gradients. These mainly correspond to the heart shadow that is usually visible in X-rays (and the generated DRRs) and the overlap with other structures, i.e., the liver, is minimal. This has improved the results in the metrics GC and GI. A further complication is that in many cases the heart shadow is faint, or blurry. This is the main reason for lower accuracy than in the agent-based approach. The agent can leverage multiple, non-hand-crafted features, does not have to rely only on the gradient information, and thus can register reasonably well even in low-quality data. It has improved the misalignment in every case. The results are promising, showing an improvement compared to current techniques. This suggests that the technique could be employed in cardiac interventions, such as CRT.

**Table 1:** TRE of the cross landmark initially (start) and after registration

	Mean	StD.	Percentiles					
			50%	60%	70%	80%	90%	100%
Start (mm)	22.80	10.50	21.42	25.22	30.03	33.50	36.96	47.88
GO (mm)	9.65	6.23	8.39	9.80	11.50	14.08	17.78	46.83
GO+ (mm)	10.49	5.97	9.42	10.87	12.49	15.00	18.54	38.02
GC (mm)	9.15	6.74	7.74	9.32	11.03	13.68	18.12	44.09
GC+ (mm)	7.80	6.30	5.91	7.51	9.30	11.55	16.43	48.37
GI (mm)	8.44	6.61	6.47	7.58	8.97	11.68	16.37	48.55
GI+ (mm)	6.79	4.75	5.63	6.50	7.48	8.84	11.77	46.14
Manual$^a$ (mm)	6.48	5.60	4.93	5.97	7.49	8.70	11.37	40.82
Agent (mm)	2.92	2.22	2.34	2.80	3.45	4.23	5.76	16.11

$^a$Manual registration for a single, randomly chosen perturbation in each case

**Fig. 5:**
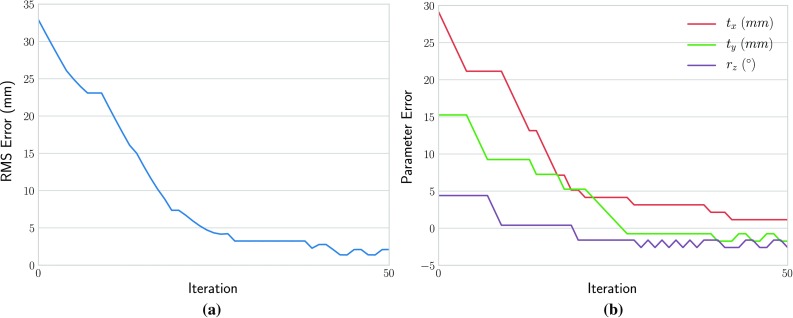
Evolution of the **a** root mean square (RMS) TRE and **b** individual parameters in the case shown in Fig. 4. The parameter error curves correspond to horizontal translation $t_{x}$ vertical translation $t_{y}$ and in plane rotation $r_z$

The evolution of the TRE and individual parameters of the agent is visualized in Fig. 5 for the case shown in Fig. 4. The TRE decreases monotonously until convergence. The figures show that a well-trained agent’s actions converge monotonously to the optimal alignment. A registration is performed within 3 s.

### Clinical CRT data

Further evaluation was performed on 19 clinical CRT datasets to evaluate registration performance in a realistic scenario. Each dataset consists of an MR acquisition and an X-ray fluoroscopy image acquired in the anterior–posterior (AP) C-arm angulation ($0\pm 5^{\circ }$ primary and $0\pm 2^{\circ }$ secondary angle). The end-diastolic X-ray frame is manually selected. Corresponding end-diastolic models were extracted from the MR images by a combination of a machine learning-based landmark detection and a minimum path algorithm based on histogram analysis [6].

An accurate ground truth registration is not available; thus, accuracy was evaluated qualitatively. Since in cardiac interventions, such as CRT, robustness of registration has priority over accuracy, the method was evaluated for robustness. After a rough, initital manual alignment, the models in the 19 cases were perturbed multiple times by in-plane, 3-DOF transformations, similarly as performed on the training data. The perturbed models were registered to the corresponding X-ray images. If the registration provides similar results for different perturbations, the method is robust. Robustness was evaluated qualitatively and quantitatively.

#### Qualitative evaluation

**Fig. 6:**
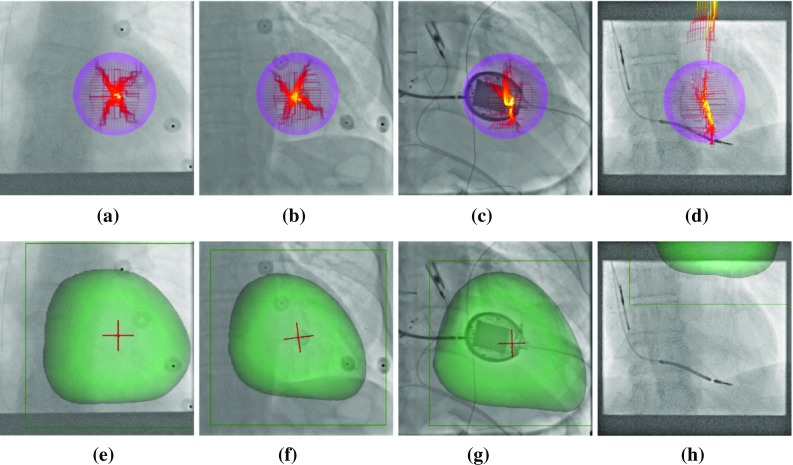
Cases showing different degrees of robustness. **a**–**d** Convergence of the center point through the agents actions from various starting positions on the boundary of the purple circle. **e**–**h** Randomly chosen exemplary results. **a** P8: highly robust **b** P12: highly robust **c** P16: robust **d** P15: least robust **e** P8: success **f** P12: success **g** P16: success **h** P15: failure

Qualitative evaluation was performed by visualizing the agents actions from the starting to the final positions. The model was perturbed 100 times from the initial alignment to generate misalignments of the center of the LV of 30 mm. The perturbed models were reregistered to the X-ray images, and the path of the center of the LV was recorded. The paths are visualized in Fig. 6 for two highly robust cases (a–b), a robust case (c) and the case showing the lowest robustness (d). A total of 15 patients showed very high robustness; the agents path has always converged to the same position. In one case, the final positions were in a less confined area, see Fig. 6c. In two cases, the agent has only diverged for a few starting positions. In the case shown in Fig. 6d, some paths are diverging (the agent has left the image), and the area where most paths end is not well constrained.

It is to be noted that the images were acquired in the standard clinical workflow; thus, they have different acquisition parameters. This results in highly varying properties, such as image quality, FOV, or resolution. Additionally, devices are often in the FOV, such as fiducial markers (Fig. 6a–b) or interventional devices, such as catheters or even an ultrasound transducer, see Fig. 6c. The registration appears to be robust against most factors, such as fiducial markers or FOV. Cases involving multiple devices (catheters, leads), or devices of larger extent (ultrasound transducer), are more challenging. The robustness is generally lower in these cases. The case of lowest robustness (patient 15) has the lowest signal-to-noise ratio and implanted electrodes. These appear to pose the main limitation in performance.

The accuracy was evaluated visually for randomly sampled results, showing the LV model overlayed on the X-ray images, see Fig. 6e–h. In cases showing robustness, the border of the overlayed LV model is aligned well with the LV shadow in the X-rays.

#### Quantitative evaluation

To measure robustness, the variance of the final registration state for different perturbations was observed. The models were perturbed on a regular grid of translations ($-30$ to $30\,\hbox { mm}$, with 5 mm sampling) with random rotations ($-\,15$ to 15), starting from a rough inititial alignment, resulting in 169 perturbations per case. The models were reregistered to the X-ray images. The median final position $\tilde{\varvec{x}}_{\varvec{f}}$ of the cross landmark was used as a reference. The L2 norms of the final positions $\varvec{x}_{\varvec{f}}$ were computed relative to this position for each dataset separately:

4 $$\begin{aligned} e_f = \Vert \varvec{x}_{\varvec{f}} - \tilde{\varvec{x}}_{\varvec{f}} \Vert _2. \end{aligned}$$

The resulting deviations $\varvec{e}_{\varvec{f}}$ show minor variance. In some patients, such as patient 15, the trajectory was diverging for perturbations at the edge of the capture range. In ten patients, there was no divergence. The patient data with the worst performance (15) had $14\%$ outliers. It is to be noted that errors above the training range (35 mm) are diverging trajectories. The median deviation was approximately 1 mm in every case. It was below 5 mm in 97.16 % of all cases, and 90 % of all deviations were below 1.42 mm.

**Fig. 7:**
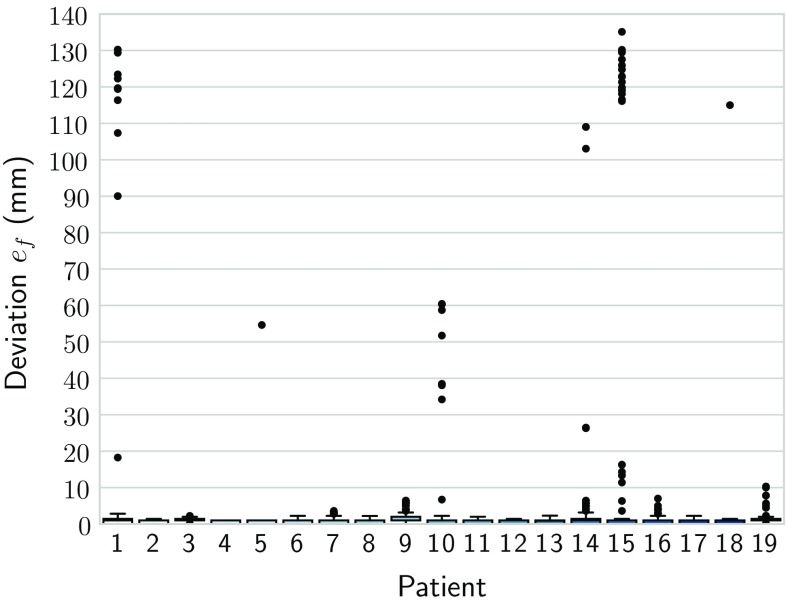
Deviations of results from the median. The points mark the outliers

## Conclusion

In this paper, a novel method for registering 3D preoperative models to 2D intraoperative images for cardiac interventions, such as cardiac resynchronization therapy (CRT), was presented. The method is agnostic to the preoperative modality, it can be, e.g., MR, CT, or ultrasound imaging, since instead of the raw image data, 3D models are registered. The models are often available from standard clinical work. To register preoperative models, i.e., the left ventricle (LV), to X-ray fluoroscopy, imitation learning was performed. The method was trained on models extracted from CT and artificial X-rays, digitally reconstructed radiographs (DRRs) (Fig. 7). It was shown that the method is robust against segmentation errors and can register LV models to DRRs with high robustness and accuracy. The trained system can be applied to other modalities, i.e., MR to X-ray fluoroscopy. The robustness and fast performance proves clinical feasibility. Furthermore, there is no interference with the standard clinical workflow: Preoperative models from clinical reporting can be used, and a single X-ray acquisition is required. Future goals are to demonstrate good performance on multiple preoperative modalities, bodyparts, and multiple C-arm angulations, making the method widely applicable in various clinical workflows.

## Electronic supplementary material

Below is the link to the electronic supplementary material.

Supplementary material 1 (mp4 16664 KB)
